# Comparative Preparation Method and Associated Cost of Lignin–Cellulose Nanocrystals

**DOI:** 10.3390/nano12081320

**Published:** 2022-04-12

**Authors:** Yi Zhang, Abu Naser Md Ahsanul Haque, Maryam Naebe

**Affiliations:** Institute for Frontier Materials, Deakin University, 75 Pigdons Road, Geelong, VIC 3216, Australia; ivf@deakin.edu.au (Y.Z.); a.haque@deakin.edu.au (A.N.M.A.H.)

**Keywords:** native and purified lignin, lignocellulose, nanocrystals, UV-shielding, hemp hurd waste, sustainable packaging

## Abstract

Lignin is a natural source of UV-shielding materials, though its recalcitrant and heterogeneous structure makes the extraction and purification processes complex. However, lignin’s functionality can be directly utilised when it stays as native with cellulose and hemicellulose in plant biomass, rather than being separated. The fabrication process of this native lignin is sustainable, as it consumes less energy and chemicals compared to purified lignin; thus, it is an economic and more straightforward approach. In this study, the properties of native and purified lignin–cellulose nanocrystals (L–CNCs) sourced from hemp hurd waste were compared to explore the differences in their morphology, UV-shielding properties and chemical structure affected by their distinct fabrication process. These two kinds of L–CNCs were further added into polyvinyl alcohol (PVA) to evaluate their reinforcement characteristics. The resulting native L–CNCs/PVA film showed stronger UV-shielding ability than purified L–CNCs. Moreover, the native L–CNCs showed better compatibility with PVA, while the purified L–CNCs/PVA interfaces showed phase separation. The phase separation in purified L–CNCs/PVA films reduced the films’ tensile strength and Young’s modulus and increased the water vapour transmission. The laboratory-scale cost of native L–CNCs production (~AUD 80/kg) was only 10% of purified L–CNCs (~AUD 850/kg), resulting in a comparatively lower cost for preparing native L–CNCs/PVA composite films. Overall, this study shows that the proposed method of production and use of native L–CNCs can be an economic approach to deliver UV-shielding properties for potential applications, such as food packaging.

## 1. Introduction

Sustainable packaging film with UV-shielding properties can help maintain food quality and provide a safer environment by reducing non-degradable plastics. Natural UV-shielding material, such as lignin, has attracted a lot of attention in the recent past [1,2]. Lignin is an aromatic polymer with inherent UV-shielding ability [3]. The UV-shielding ability of lignin is originated from its aromatic structure and presence of chromophoric groups, such as catechols, aromatic ketones, coniferaldehyde, stilbenes and conjugated phenolics [4]. In nature, it is abundant, though complexly synchronised with cellulose and hemicellulose in most plant-based resources. Though it can be a good source of sustainable UV-shielding filler, its recalcitrant and heterogeneous structure makes the separation process (from cellulose and hemicellulose) difficult. The purification of lignin, thus, requires harsh chemical treatments [5]. However, the functionality of lignin can also be utilised as native lignin when it coexists with cellulose and hemicellulose, bypassing the chemically harsh separation.

Furthermore, studies [6] show that the tensile strength of native lignin–cellulose nanocrystals (L–CNCs) is significantly higher than purified L–CNCs, as the original bonding between lignin and cellulose is retained. Thus, native lignin is believed to reduce the associated cost of preparation (due to less intensive chemical treatment) compared to purified lignin, while also delivering superior properties. However, to justify this hypothesis, a comparative analysis of the UV-shielding performances between native L–CNCs and purified L–CNCs collected from the same source is rare.

Only a recent study by Teh et al. [7] reported a comparative analysis of native L–CNCs and purified L–CNCs collected from oil palms. The findings are encouraging since the energy cost of native L–CNCs was found to be 80% less compared to the purified L–CNCs. However, the study was mainly focused on the method development, though the UV-shielding performance and suitability of practically applying those L–CNCs inside any polymer were not investigated. Moreover, a comparative study on the native and purified L–CNCs sourced from a more universal resource than the oil palm is necessary.

Hemp is a multi-use crop with around 6000 years of history across the world and is treated as one of the fundamental agricultural plants. Hemp has varied applications, including in textiles, food, paper, medicines, cosmetics and pharmaceuticals, and, currently, above 25,000 types of hemp products exist in the global market [8]. However, hemp hurd is discarded as a low-value waste and often landfilled after industrial processing [9]. Fabricating L–CNCs from hemp hurd is economic and sustainable, given the abundance of hemp around the globe and possible effective utilisation of its waste [10]. In addition, hemp hurd contains a large amount of lignin compared to some common lignocellulose materials (such as rice straw and corn stover) [11], and 53% of the phenolic group from lignin is sinapyl alcohol, which is the most effective UV-shielding group of lignin [12]. Hence, aside from its economic potential, hemp hurd is likely to deliver superior UV-shielding benefits.

Though preparation of cellulose nanofibers accompanied with native lignin has been previously reported from hemp [13], our group is the first to report the fabrication of nanocrystals, i.e., native L–CNCs from hemp hurd [14]. We found that the UV-shielding ability of the fabricated native L–CNCs from hemp hurd is comparable to the traditional UV-shielding film made of ZnO/lignin/polyurethane when we used the native L–CNCs with a ratio of 20 wt.% in polyvinyl alcohol (PVA). However, we only reported the effectiveness of native lignin–nanocellulose/PVA composite and did not compare with purified L–CNCs. Mendoza et al. [15] grafted phenolic esters into CNCs as UV-shielding filler for a PVA matrix, while using a complex reaction (click-type copper-catalysed azide/alkyne cycloaddition reaction) and a toxic chemical (propargyl bromide). Espinosa et al. [16] found the UV-shielding ability of the native lignin–nanocellulose/PVA composite to be better than that of nanocellulose/PVA composite; the comparison was only between the one with lignin and without lignin. Overall, the focus of the previous studies was on the materials fabrication rather than the qualitative comparison. Therefore, there is a lack of understanding on the UV-shielding performance and reinforcing compatibility of native L–CNCs relative to the purified L–CNCs, which requires attention.

Considering the possible advantages of using native L–CNCs over purified L–CNCs, as well as the limited studies involving a comparative analysis focusing on UV-shielding performance, this study aims to provide a comprehensive insight into the differences in performance and preparation cost of native and purified L–CNCs independently, as well as after the reinforcement, and further analyse their relative compatibility inside the polymer matrix. To achieve that, we prepared both native and purified L–CNCs from hemp hurd, using a ball-milling-assisted extraction process and later compared their UV-shielding properties before and after the reinforcement. We also observed their compatibility inside the matrix and compared the overall production cost. We chose hemp hurd as the source, due to its worldwide availability, which is wider than the previously reported resource (oil palm), and its lignin-rich content. We chose polyvinyl alcohol (PVA) as the polymeric matrix, which is widely used as a matrix for nanocellulose reinforcement in the food-packaging area, due to its good film-forming properties [17]. Overall, the differences in the L–CNCs regarding the morphology, zeta potential, UV-shielding efficiency and their influences on the tensile and water-vapour transmission properties are reported, and relevant costs are compared.

## 2. Materials and Methods

### 2.1. Materials

Hemp hurd was sourced from the Commonwealth Scientific and Industrial Research Organisation (CSIRO, Geelong, Australia). Sodium sulphite (anhydrous, 98–100.0%) was purchased from Sigma-Aldrich, Melbourne, Australia; sodium hydroxide (≥97.0%, pellets) and sulphuric acid (98 wt.%) were purchased from Chemsupply (Adelaide, Australia). Poly (vinyl alcohol) solution (PVA) 5 wt.% was purchased from Flew Solutions, Brisbane, Australia. The molecular weight (Mw) of the PVA was up to 100,000, and the degree of hydrolysis was 87–89%.

### 2.2. Preparation of Native and Purified Lignin–Cellulose Nanocrystals (L–CNCs)

The hemp hurd was milled into particles by a two-step milling process [18]. The hemp hurd was briefly cut into coarse particles with a cutter milling (19 Universal, Fritsch Pulverisette, Idar-Oberstein, Germany) and then milled into fine particles by using an Attritor mill (2S, Union Process, Akron, OH, USA). The preparation method of native lignin–cellulose nanocrystals (L–CNCs) was similar to our previous study [14]. Briefly, the 4 g hemp solution, which contained 0.08 g hemp hurd, was added, along with 64 wt.% sulphuric acid, to a round-bottom flask and hydrolysed at 45 °C for 7 h. To the solution was added ten-fold DI water to quench the reaction, and the solution was later centrifuged at 6000 rpm for 15 min, at 4 °C, to remove extra acid. The solution was then sonicated and stored in a glass reagent bottle.

For purified lignin–cellulose nanocrystals (L–CNCs), first lignin was extracted from hemp herd and then was mixed with cellulose nanocrystals obtained from the hemp hurd. The raw material amount was the same as the native lignin–cellulose nanocrystals. Briefly, the hemp hurd powder was dissolved in an alkaline solution, a mixture of 2.5 mol/L NaOH and 0.4 mol/L Na_2_SO_3_ in DI water [19]. After being kept boiling for 12 h, the lignin was extracted from hemp hurd powder and remained in the alkaline solution. The bleaching method was not used, as it could destroy the chromophore structure of Kraft lignin [20]. The filtered solution was considered as purified lignin (Kraft lignin). The sediment was further hydrolysed with 25 mL of 64 wt.% sulphuric acid for 1 h at 45 °C to produce cellulose nanocrystals [21]. The purified lignin solution was then mixed with the prepared cellulose nanocrystals.

### 2.3. Preparation of L–CNC/PVA Film

The same volume of L–CNCs solutions was mixed with PVA solution at the ratio of 5 wt.% (calculated by initial hemp weight) and cast in the Petri dish. The weight of PVA of each sample was the same. The casting solution was dried in the fume hood for at least 12 h, and the thickness of L–CNCs/PVA film was measured by the Digital Caliper (Meinaite, Shanghai, China).

### 2.4. Characterisations

#### 2.4.1. Morphology

The Atomic Force Microscopy (Asylum Research, Santa Barbara, CA, United States) was used to image the lignin–cellulose nanocrystals at a range of 5 × 5 μm^2^ area with a resolution of 256 × 256 pixels from a Cypher AFM in air. The testing solution was first diluted 1000 times and sonicated for 5 min. Then the solution was dropped on the silicon wafer and dried in the fume hood overnight. The length and height were measured by choosing 30 random particles. The width was not measured, as some aggregates may be hidden in the lateral direction, causing inaccurate results [22]. Thus, the aspect ratio was calculated by the length/height (diameter), and the height measured from AFM was recognised as the diameter.

The transmission Electron Microscopy (TEM) technique was used to obtain the morphology, and microstructure images of purified L–CNCs and native L–CNCs were captured by using a JEOL 2100 LaB6 (Jeol, Akishima, Tokyo, Japan) at an accelerating voltage of 80 kV.

#### 2.4.2. Chemical Structure

The change in chemical structure of L–CNCs was measured by the KBr pellet method through the transmission mode. The pellet was a mixture of 200 mg KBr and 1 mg L–CNCs. The transmission data were transferred into the absorption data by using the following equation.

A= 2 − log(T%)

where A is absorption, and T is the transmission.

The change in the structure of purified L–CNCs/PVA and native L–CNCs/PVA was confirmed by the Attenuated Total Reflection Fourier-transform infrared spectra (ATR-FTIR) from Vertex 70 spectrometer (Bruker, Billerica, MA, USA) from 600 to 4000 cm^−1^ wavenumbers at a 4 cm^−1^ scan resolution.

#### 2.4.3. Absorbance and Transmittance

The UV absorptions of the solution were measured by Cary 300 UV–Visible Spectrophotometer (Agilent Technologies, Santa Clara, CA, USA) from 200 to 400 nm, and the UV transmission of the films was measured by using a Cary 5000 UV–Vis–NIR spectrophotometer (Agilent Technologies, Santa Clara, CA, USA), from a 200 to 800 nm wavelength. The ultraviolet protection factor (UPF) value was measured by the YG902 UPF spectrophotometer (Fanyuan Instrument Co., Ltd., Hefei, China), according to the AS/NZS 4399 standard. The obtained values were further normalised by the film thickness.

#### 2.4.4. Tensile Properties

The maximum stress at break, Young’s modulus and elongation at break were measured by using a Universal Instron tensile testing machine (Instron, Norwood, MA, USA) with a 100 N load cell. The gauge length was maintained at 10 mm × 10 mm. Five specimens of each sample were tested, and averages with standard deviations were reported. The stress was calculated by divided the force to the volume of film; thus, different thicknesses were considered. The film thickness influencing the Young’s modulus was also considered, as it is the slope of the strain–stress curve in the linear part.

#### 2.4.5. Water-Vapour Properties

The films’ water-vapour transmission rate and water-vapour permeability were tested using a W3/031 water-vapour transmission rate tester (Labthink, Jinan, Shandong, China) at 38 °C in temperature and 90% relative humidity, according to TAPPI T 464 [23].

#### 2.4.6. Statistical Analyses

The statistical analyses were performed by a one-tailed t-test between the datasets by using IBM SPSS (Chicago, IL, USA). When *p* ≤ 0.05, this indicates that the reported data are significantly different from each other, and when *p* > 0.05, the data have no significant difference.

## 3. Results and Discussion

### 3.1. Morphology

**Figure 1:**
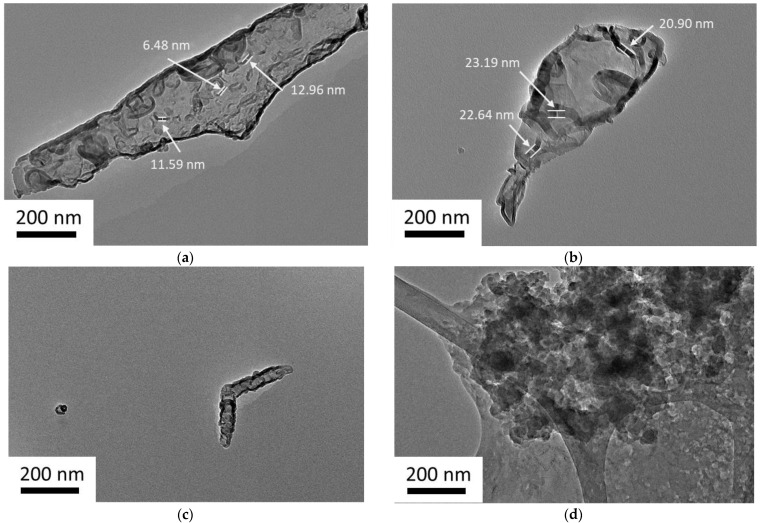
TEM images of purified L–CNCs in (**a**,**c**) and native L–CNCs in (**b**,**d**).

The morphologies of purified L–CNCs and native L–CNCs at nano-level are shown in Figure 1. The width of the purified L–CNCs was smaller than that of the native L–CNCs, as shown in Figure 1a,b. After mixing it with CNCs, the purified lignin formed light grey pellicles covering the CNCs, and these pellicles hold the CNCs together. This observation is similar to that reported by Dima et al. [24]. The purified L–CNCs showed a trend toward generating thinner L–CNCs, because most of the lignin was extracted and not included in the acid hydrolysis procedure. For the native L–CNCs samples, since lignin exists and acts as an obstacle for hydrolysis of cellulose and hemicellulose, the acid hydrolysis procedure will be harder than purified L–CNCs, and, therefore, larger native L–CNCs are formed.

It was also observed that the purified L–CNCs with a rod-like structure aggregated together, and one single particle was observed in Figure 1c. This single particle could be only CNCs, and not attached to the purified lignin, since the morphologies observed in this study were mostly similar to the morphology of pure CNCs reported in the literature [25]. This indicates that the connection between purified lignin and CNCs are weak. The literature suggested that the weak linkage between lignin and CNCs can be improved by a cross-linking agent, such as glutaraldehyde [26]. In case of the native L–CNCs, the size of the particles was probably broken down by the hydrolysis, while some particles were entangled. However, in some other areas, particles with irregular shapes were observed. The native L–CNCs showed an uneven distribution related to the lignin nanoparticles aggregation, as shown in Figure 1d. The longer acid hydrolysis process removed some of the amorphous areas, while some lignin nanoparticles were exposed and mixed with L–CNCs [7].

**Figure 2:**
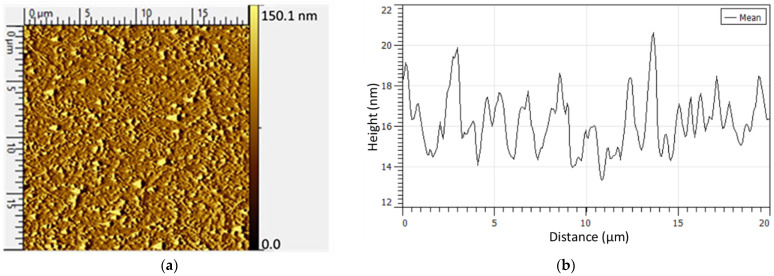

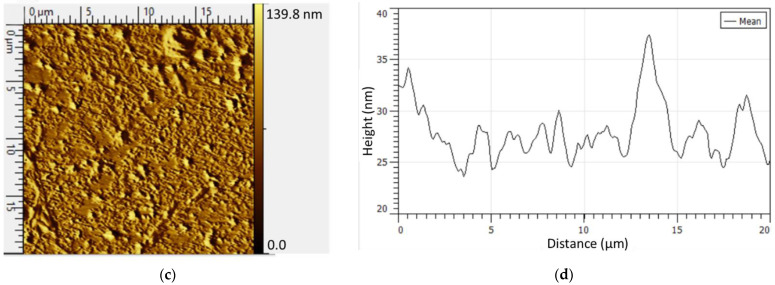
AFM images of (**a**) purified L–CNCs and (**c**) native L–CNCs; (**b**) Z-profile (height) of (**a**); (**d**) Z-profile of (**c**).

The larger scale (at the micro-level) distribution of L–CNCs can be observed from the AFM images shown in Figure 2. The size of purified and native L–CNCs is listed in Table 1. To obtain a similar length of L–CNCs, and for fabricating the purified L–CNCs, after extracting the lignin (12 h extraction time), only 1 h of hydrolysis time was performed to produce cellulose nanocrystals from the sediment separated from lignin. Meanwhile, this time for native L–CNCs was 7 h. However, the whole fabrication process of purified L–CNCs (total of 13 h) took more time compared to the fabrication of native L–CNCs (around 7 h). This may indicate that lignin in native L–CNCs protects cellulose against depolymerisation [27]. The native L–CNCs showed a higher aspect ratio (~30), whereas purified L–CNCs only reached almost half (~16.6). The higher aspect ratio of native L–CNCs could be related to the longer hydrolysis time (7 h for native L–CNCs against 1 h for purified L–CNCs). A longer acid treatment could decompose cellulose into sugar, thus decreasing the diameter (height). This can also be confirmed from the yield of native L–CNCs (~14%), as it is much lower than what has been reported in the literature (50%) [7]. The yield of purified L–CNCs was over 100%, which could relate to the extraction method of lignin. In the lignin extraction process, the intermolecular ester bonds between lignin–cellulose and lignin–hemicellulose are broken down and phenolic hydroxyl groups, carboxyl groups and suphonate group are introduced into the phenylpropane unit of lignin [28]. Hence, the decrease of amorphous cellulose amount was less than the increase of lignin amount. Thus, the yield of purified L–CNCs was over 100%. The native L–CNCs also showed rod-like structures, similar to those of the CNCs in the purified L–CNCs in Figure 2a.

The zeta potential of both purified L–CNCs and native L–CNCs was tested at pH = 7, and the difference between them was statistically significant. The zeta potential of L–CNCs was less negative than those reported in the literature as the high dilution of the solution [29]. Both purified L–CNCs and native L–CNCs carry sulphonated groups, which probably have led to a negative surface charge. The native L–CNCs showed more negative charge due to the higher sulphonation degree and it is similar to those reported in Aro et al. study [30].

### 3.2. Absorption Properties

**Figure 3:**
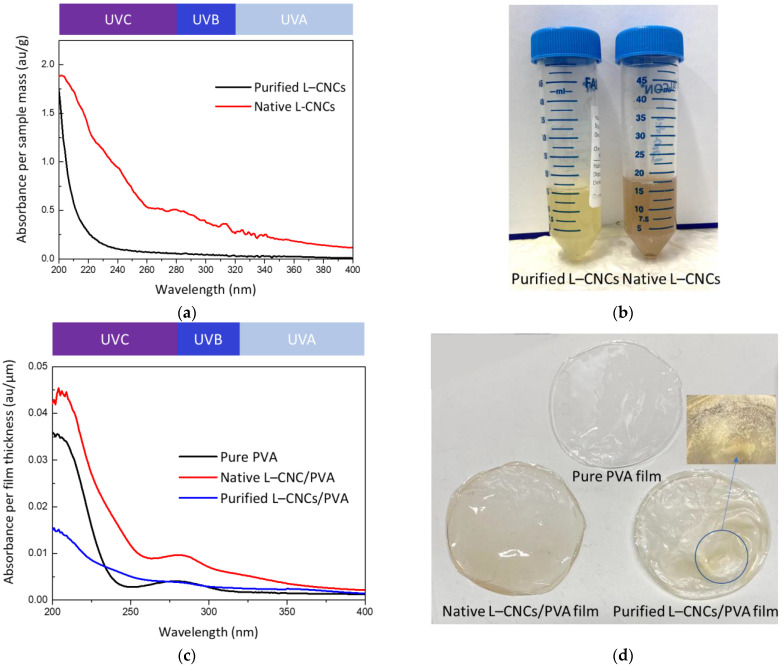
UV absorption (**a**) and a digital image of (**b**) purified L–CNCs and native L–CNCs solution, (**c**) the UV absorption (normalised by the film thickness) and a digital image of (**d**) of purified L–CNCs film and native L–CNCs film. The black background was used to enhance the contrast, while showing the enlarged part of the purified L–CNCs/PVA.

Though the CNC is normally known to absorb UV-light, the purified lignin is. Thus, the difference between purified L–CNCs and native L–CNCs is worth studying. The UV absorption of purified L–CNCs and native L–CNCs is shown in Figure 3a, and the digital image of these two solutions is shown in Figure 3b. At around the 200–400 nm wavelength range, the native L–CNCs showed a stronger absorbance than the purified L–CNCs. This is probably because of the difference in the treatment process. As observed from the zeta potential value, the sulphonation degree of native L–CNCs was higher than that of the purified L–CNCs. The sulphuric acid hydrolysis can change the structure of the native lignin by cleaving the β-O-4 and β-β bonds and increasing the quantities of phenolic hydroxyl groups, carboxylic acids and carbonyl groups [31]. These chromophore groups lead to the increase of the UV-shielding ability in the native L–CNCs. A similar observation was also found in our previous study [14]. Though the yield of native L–CNCs was low, the generation of more chromophore groups can be confirmed by the red-brown colour perceived from native L–CNCs (Figure 3b) [7]. The main peak at 280 nm that was observed in the native L–CNCs sample was mainly related to the lignin, as pure PVA has a weak peak in that wavelength.

The UV absorption of films and their digital images are shown in Figure 3c,d. Though identical volumes of L–CNCs were added to the PVA matrix, the yield of L–CNCs was different among native L–CNCs and purified L–CNCs samples. This resulted in fabricated films with different thicknesses. The thicknesses of pure PVA, purified L–CNCs/PVA and native L–CNCs/PVA were 31 ± 14 μm, 106 ± 41 μm and 40 ± 8 μm, respectively. Thus, the UV absorbance was normalised by the film thickness. As the PVA weight was kept constant, the difference in UV absorption of the film was mainly related to the fillers. The Klason lignin of native L–CNC was similar to the hemp particle form our previous study [14]. The purified L–CNCs was also from hemp particle, and lignin was not removed. Thus, we assume the Klason lignin was similar in both native and purified L–CNCs. Therefore, after normalisation, the difference of UV-shielding properties is based on the lignin treatment method. Moreover, the UV absorption of composite films was found to be similar to the L–CNCs solutions. The UV-shielding properties were largely led by the existence of lignin in both L–CNCs samples. The UV absorption at 280 nm was observed on both pure PVA and L–CNCs/PVA samples, and the absorption of native L–CNCs/PVA was double that of pure PVA and purified L–CNCs/PVA. The absorption of pure PVA showed a minor peak at the wavelength of 280 nm, which also confirmed the minor influence of pure PVA on UV-shielding property. The native L–CNCs/PVA film showed a stronger UV-shielding property due to the native lignin. In the fabrication process, native lignin generated more chromophore groups, increasing UV-shielding ability and darkening the colour. Though the purified L–CNCs/PVA sample showed a stronger UV-shielding property than the pure PVA at 300–400 nm, it was much lower than that of the native L–CNCs/PVA.

Moreover, as shown in Figure 3d, the distribution of purified L–CNCs was uneven in the PVA matrix, and some parts of the film were shrinking. As shown in Table 1, there is a large difference in L–CNCs yields after hydrolysis of hemp (almost 118% for purified L–CNCs and 14% for native L–CNCs). Therefore, the shrinking of the film could be caused by high ratio of purified L–CNCs in PVA. The high ratio of purified L–CNCs could increase the chance of particle agglomeration. This resulted in a large deviation in the UV-shielding ability of this sample. It was also confirmed by the T% data and the normalised UPF number presented in Table 2. UV radiation is divided into UVC (200–280 nm), UVB (280–320 nm) and UVA (320–400 nm), according to the wavelength. Most of the UVC radiation is absorbed by the ozone layer; thus, it is not a concern for food degradation. In this study, both native L–CNCs/PVA and purified L–CNCs/PVA showed better UV-shielding performance in the UVB range and the main UV absorption peak of lignin in the UVB range. As mentioned earlier (Materials and Methods), the obtained UPF value was normalised by the film thickness. Comparing with the pure PVA and purified L–CNCs/PVA, the normalised UPF value of native L–CNCs/PVA was higher. This is consistent with the UV absorption of purified and native L–CNCs, as shown in Figure 3. The deviation of the UPF number of purified L–CNCs/PVA was over 50% of the normalised UPF number. This could be related to the phase separation and immiscibility of lignin and PVA [32]. The phase separation was also observed in our several laboratory trials, as some of the purified L–CNCs samples remained in the Petri dish and did not peel off. This phase-separation behaviour of purified L–CNCs/PV that might be related to the high ratio of purified L–CNCs in PVA is likely to affect the compatibility of the filler and matrix and, thus, may influence the overall mechanical properties.

### 3.3. Chemical Structure

**Figure 4:**
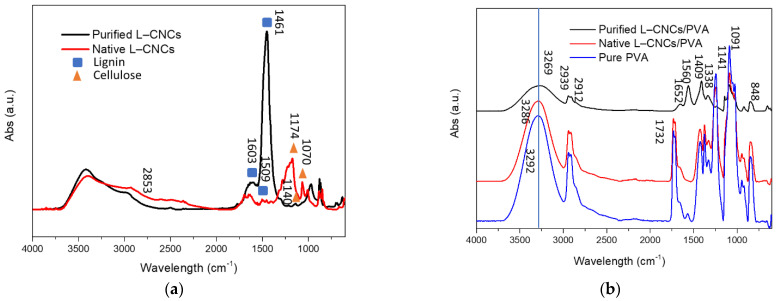
FTIR spectra of (**a**) purified L–CNCs and native L–CNCs, (**b**) purified L–CNCs/PVA, native L–CNCs/PVA and pure PVA.

FTIR spectroscopy was used to characterise the structural changes of LCNCs and L–CNCs/PVA films, and the result is shown in Figure 4. As shown in Figure 4a, the peak at 2853 cm^−1^ corresponds to the methoxy group [33,34]. The native L–CNCs showed a stronger peak than that of the purified L–CNCs at 2853 cm^−1^, which could response to the stronger UV-shielding ability of lignin. As methoxy group, an electron-donating group, can contribute to the conjugated system in lignin [3]. The purified L–CNCs showed a strong peak at 1461 cm^−1^, which was related to the C–H deformation (asymmetric in –CH_3_ and –CH_2_) [35], rather than a UV-functional group. As shown in Figure 4b, the spectra at the 3269, 3286 and 3292 cm^−1^ were corresponding to the –OH stretching vibration of the alcoholic hydroxyl group and phenolic hydroxyl from PVA and L–CNCs [36]. This indicates that the hydrogen bond between native L–CNCs and PVA is different from that of purified L–CNCs and PVA. No significant change was observed from the spectra of native L–CNCs/PVA and pure PVA. This indicates that only the hydrogen bond was probably formed between native L–CNCs and PVA. The peak at 1732 cm^−1^ represented the C=O vibrations from acetate functional groups of partially hydrolysed PVA [37]. The acetate group was transformed into –OH group under the presence of NaOH in purified L–CNCs. The peak at 1652 cm^−1^ was attributed to the C=O stretching in α, β-unsaturated aldehydes or ketones [38] of purified L–CNCs. The structure of lignin changed after the alkaline extraction. The peak at 1560 cm^−1^ was attributed to the aromatic ring vibrations [38]. This peak was observed in purified L–CNCs/PVA, but not found in the native L–CNCs/PVA sample. It is consistent with the fact that the initial UV-shielding ability of purified L–CNCs/PVA was stronger than the native L–CNCs/PVA and pure PVA. However, after considering the thickness of the film, the UV-shielding ability was reduced. The peak at 1409 cm^−1^ was assigned as C–C stretching vibrations [39]. The peak at 1338 cm^−1^ possibly resulted from the C–O stretching of the syringyl ring of lignin. The peak at 1091 cm^−1^ was attributed to the C–O–C stretching, and 848 cm^−1^ was assigned to C–C stretching of PVA.

Overall, the FTIR result confirmed the changes in the structure of purified lignin. Moreover, the hydrogen bonding perceived between L–CNCs and PVA matrix is likely to influence the mechanical properties of the composites, as is discussed in the next section.

### 3.4. Tensile Properties

**Figure 5:**
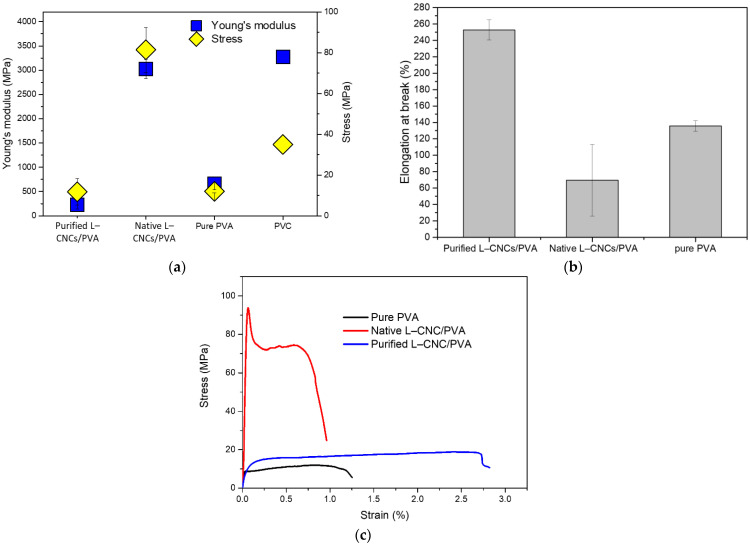
(**a**) Young’s modulus, maximum stress at break, (**b**) elongation at break and (**c**) a representative stress–strain curve of purified L–CNCs/PVA and native L–CNCs/PVA film. The data of PVC were adopted from Reference [43], and the data of pure PVA were from Reference [18].

The Young’s modulus, maximum stress at break and elongation at break of pure PVA, purified L–CNCs/PVA and native L–CNCs/PVA films are shown in Figure 5a,b. Since the variation in film thickness was considered in Young’s modulus and stress calculation, the calculated values are comparable for each film. The native L–CNCs/PVA film showed a higher Young’s modulus and higher maximum stress at break than the purified L–CNCs/PVA film and pure PVA. The higher aspect ratio of native L–CNCs could contribute to the increase in Young’s modulus [40]. After adding L–CNCs to PVA, the Young’s modulus of both purified L–CNCs increased, and the Young’s modulus of native L–CNCs/PVA film (3023 MPa) was found to be comparable to commercial polyvinyl chloride (PVC) food-packaging film (cling wrap), as shown in Figure 5a. The low Young’s modulus of purified L–CNCs could be related to its uneven distribution.

No significant differences (*p*-value = 0.983 > 0.05) was observed for the maximum stress at break of purified L–CNCs/PVA and pure PVA, though the maximum stress at break of the native L–CNCs/PVA sample increased to almost eight times than that of purified L–CNCs/PVA and the PVC [41]. The large standard deviation of purified L–CNCs/PVA is related to the unevenness of the film. The higher maximum stress of native L–CNCs could be related to its uniform desperation in PVA. As previously discussed (Figure 3d), the native L–CNCs showed a good dispersion in the PVA, which probably led to a uniform stress transfer on the filler/matrix interfaces [36]. However, due to the phase separation, the compatibility between purified L–CNCs and PVA was probably not good enough, thus resulting in poor distribution of L–CNCs inside PVA and lower maximum stress at break than that of native L–CNCs/PVA.

The elongation at break is shown in Figure 5b,c; the native L–CNCs/PVA films were reduced compared to the pure PVA and purified L–CNCs/PVA. The native L–CNCs fillers were responsible for the decrease of elongation at break. This is because the addition of native L–CNCs interrupted the PVA mobility [42]. However, despite the reduction in elongation property, the native L–CNCs/PVA films retained enough flexibility and showed closer value to the commercial PVC cling wrap. The purified L–CNCs/PVA showed extended elongation, which could relate to its thickness. The film is thicker than the pure PVA and native L–CNC/PVA, thus increasing the elongation.

### 3.5. Water-Vapour Properties

**Figure 6:**
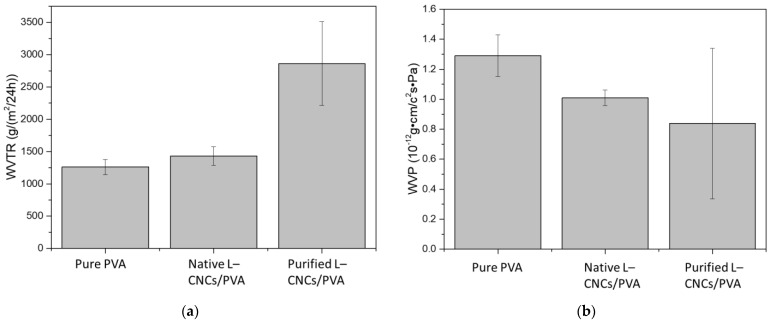
(**a**) Water-vapour transmission rate (WVTR) and (**b**) water-vapour permeability (WVP) of pure PVA, purified L–CNCs/PVA film and native L–CNCs/PVA film.

The water-vapour transmission rate (WVTR) and water-vapour permeability (WVP) of PVA and L–CNCs/PVA films are shown in Figure 6. The pure PVA and native L–CNCs films showed similar water-vapour transmission rates, while the purified L–CNCs/PVA film showed a 2-time increase. This is probably related to the alkaline treatment of lignin that changed the original lignin structure and affected its interaction with PVA. The changes in lignin structure were also confirmed from the FTIR result discussed above. Moreover, the poor interaction (less compatibility) probably created more vacant spaces on the interfaces of the purified L–CNCs and PVA, thus letting more water vapour pass through. The native L–CNCs/PVA did not show significant change compared with pure PVA. This could be related to the low yield of the native L–CNCs sample and a good interaction (compatibility) between native L–CNCs and PVA. Thus, a uniform distribution provided a barrier against the water vapour by the possible hydrogen bonding, as observed from the FTIR result. Therefore, even though the average vapour transmission rate was slightly higher than the pure PVA film (probably due to negligible vacant areas by reinforcement), the difference was not statistically significant. The difference between WVTR and WVP is because the WVP considers the thicknesses of the film and vapour passed that were divided by film thickness [44]. As the film thickness is different, the thicker film could restrict more vapour from the transmission. Though the purified L–CNCs/PVA sample showed good permeability, the reason could relate to the high-yield purified L–CNCs/PVA. Under the same thickness, the film contains more particle to restrict the vapour. However, the weight of the film would be different if the film has the same thickness.

### 3.6. Cost Estimation

The cost estimation is important for the commercial usage of food-packaging film. To provide an insight into the production cost of native and purified L–CNCs, and the composite films, the expenses at each stage were calculated and are listed in Table 3. As the hemp hurd is a waste material and can be collected at zero or negligible cost, the cost for hemp hurd was not considered.

Overall, the cost of purified L–CNCs fabrication was found to be ten times higher than that of native L–CNCs. This was because the fabrication of native L–CNCs did not require a lignin extraction process. This result is consistent with the recent study of Teh et al. [7], wherein the cost of native L–CNCs production was reported to be 62% lower than that of purified L–CNCs and required 80% less energy than purified L–CNCs. Our study showed a slightly higher cost for production of L–CNC compared to Teh et al.’s study [7], due to the cost of the different chemicals used in the extracting process. However, Teh et al. did not include the cost of sulphur dioxide that was presumably used for lignin removal and which would increase the cost of production.

In the current study, though the ball-milling process largely increased the electricity cost of both L–CNCs samples, the total cost of production for both L–CNCs/PVA composites will be much lower if bulk production is carried out. Furthermore, the power (Watts) of the machines used in the calculation considered their maximum capacity, though the actual power consumption and electricity cost were likely to be lower than the reported value.

The cost of chemicals was the main part of the cost for the fabrication of L–CNCs. However, it is worth mentioning that the industry-scale cost will be much lower than these, due to the reduced price of bulk chemicals. Moreover, the cost of the film was evaluated by the 5 wt.% addition of L–CNCs; thus, the cost of L–CNCs/PVA composite mainly depended on the PVA as it contributed 95 wt.%. The total cost of the L–CNCs/PVA composite can be reduced more if L–CNCs increase.

## 4. Conclusions

In this study, the UV-shielding property of native and purified lignin integrated CNCs was compared after collection from hemp hurd. The L–CNCs samples were further added to the PVA matrix, and the properties of L–CNCs/PVA film were evaluated for potential application in food-packaging film. The native L–CNCs reached a similar length of purified L–CNCs after a longer hydrolysis time, due to the coexisting of lignin and hemicellulose. However, native L–CNCs showed a stronger UV-shielding efficiency than that of purified L–CNCs, due to the generation of more chromophore groups. The purified L–CNC/PVA film showed phase separation and poor compatibility with PVA, which led to an unstable UV-shielding ability. Compared to the purified L–CNCs/PVA film, the tensile properties of native L–CNCs/PVA were closer to the commercial packaging products, such as PVC cling wrap. Moreover, the lower cost of native L–CNCs/PVA preparation made it a promising candidate as a UV-shielding food-packaging material.

## Figures and Tables

**Table 1:** Size and zeta potential of purified L–CNCs and native L–CNCs.

	Length (nm)	Height (nm)	Aspect Ratio	Yield (%)	Zeta Potential (mV)
Purified L–CNCs	465.3 ± 159.5	27.99 ± 2.68	16.6	117.5	−7.6 ± 5.5
Native L–CNCs	485.3 ± 172.3	16.18 ± 1.365	30.0	13.8	−24.5 ± 4.5

**Table 2:** Transmittance and normalised ultraviolet protection factor (UPF) of pure PVA, native L–CNCs/PVA and purified L–CNCs/PVA.

	T_280nm_% *	T_400nm_%	T(UVA)%	T(UVB)%	Normalised UPF
Pure PVA	76.02 ± 3.65	91.41 ± 0.02	91.41 ± 0.74	87.24 ± 2.83	32.82 ± 0.77
Native L–CNCs/PVA	41.17 ± 4.58	82.17 ± 0.95	62.83 ± 3.43	41.11 ± 4.31	50.38 ± 4.94
Purified L–CNCs/PVA	26.44 ± 15.86	55.89 ± 19.03	31.14 ± 13.41	23.90 ± 11.60	42.34 ± 23.42

* For easy understanding, the T% is not normalised; T_280__nm_% means the UV transmission in the 280 nm; and T(UVA)% means the UV transmission in the range of 320–400 nm. The UPF is normalised by film thickness.

**Table 3:** Approximate cost to produce purified L–CNCs/PVA and native L–CNCs/PVA composite films on a laboratory scale.

	Purified L–CNCs				Native L–CNCs			
Electricity *	kW	h	kW-h	Cost AUD/kg	kW	h	kW-h	Cost AUD/kg
Mechanical milling								
Cutting	5	0.05	0.25	0.05	5	0.05	0.25	0.05
Attrition	2.24	20	44.8	8.72	2.24	20	44.8	8.72
Magnetic stir	0.6	1	0.6	0.12	0.6	7	4.2	0.82
Centrifuge	1.05	1	1.05	0.20	1.05	1	1.05	0.20
(a) Total cost for electricity				9.09				9.73
Chemicals for L–CNCs								
NaOH				18.72				
Na_2_SO_3_				814.98				
H_2_SO_4_				8.52				72.54
(b) Total cost for chemicals				842.22				72.54
(a + b) Total cost for L–CNCs				851.31				82.27
Composite films								
PVA				132.53				132.53
L–CNCs (5 wt.%)				42.57				4.11
Total cost for 5 wt.% film (per/kg)				166.75				130.13

* Electricity cost (AUD/kg) (AUD 0.1946/(kwh) in 2021, Victoria, Australia [45]). The electricity cost varies from different countries.

## Data Availability

Not applicable.
